# The Influencing Factors of Haze Tolerance in China

**DOI:** 10.3390/ijerph16020287

**Published:** 2019-01-21

**Authors:** Lingyi Zhou, Yixin Dai

**Affiliations:** School of Public Policy and Management, Tsinghua University, Beijing 100084, China; ly-zhou14@mails.tsinghua.edu.cn

**Keywords:** haze, risk perception, environmental risk management, tolerance

## Abstract

Haze pollution has become the most serious environmental risk in China and generated a large amount of public concerns. Influencing almost all the citizens in the polluted area, it is necessary and important to take public perception as an essential element in haze abatement. From the perspective of social psychology, this paper explores haze tolerance in Beijing, Shanghai, and Guangzhou, also the key influential factors on haze tolerance from four dimensions: political trust, perceived risk, cost perception, and haze knowledge. Based on the sample of 517 respondents, the results show that compared with Shanghai and Guangzhou, Beijing residents had the lowest tolerance level of haze pollution but have the highest levels of trust in the government’s capacity to control haze and self-evaluation of their own haze knowledge. People in Shanghai had the lowest cost perception and the strongest willingness to acquire haze knowledge. Meanwhile, the empirical analysis revealed that political trust and cost perception could enhance the public’s haze tolerance while perceived risk and haze knowledge had negative impacts on tolerance. Also, our research could provide some suggestions to government officials when making policies for abating haze pollution from the perspective of social risk control. Policy makers are supposed to launch various policy instruments to control haze effectively and engage the citizens in the decision-making process to improve their political trust, and publicize the knowledge of haze pollution to help the public to acquire objective and scientific knowledge and diminish unnecessary worries.

## 1. Introduction

Haze has become the most serious and concerning environmental risk in China. According to the 2016 Report of Yale University’s Environmental Performance Index, air quality of China is the penultimate in the world, and the national annual average concentration of PM_2.5_ ranks last in the world [1]. China has become the worst-hit area of PM_2.5_ pollution. Previous studies have shown that atmospheric particulate pollution ranks fourth among the 20 major factors causing fatal harm to the public in China [2]. Haze pollution can aggravate respiratory and cardiovascular diseases and even lead to cancer or premature death [3]. Chen et al. [4] found that the cities north of the Huai River were prone to severe haze during the winter heating period, thus reducing the average life expectancy of 500 million residents by 5.5 years per person. At the same time, the rise of civic awareness and the rapidity of information dissemination have posed new challenges to the risk management of haze. In January 2013, haze crisis impacted over 30 provinces across the country four times. Since then, the keyword “haze” has attracted more and more public attention [5].

Environmental risk is the probability of occurrence and consequences of unfortunate events caused by spontaneous natural causes and human activities, which can cause damage or even destruction to human society and the natural environment. The primary goal of environmental risk management is to control environmental risks to a socially and naturally acceptable level to avoid serious consequences [6]. At present, most scholars have studied environmental risks of haze in China from the perspective of environmental science, such as physical and chemical conditions of haze, its causes, and its harm to social and economic development [7,8,9,10,11]. Little attention has been paid to the human and social factors related with environmental risks, such as public perception of haze risks and how public perception affects the design and implementation of haze control policies. Based on the data collected in Beijing, Shanghai, and Guangzhou through a questionnaire survey, we adopted an ordinal logistic model to explore the influencing factors of haze tolerance from four dimensions, that is, political trust, risk perception, cost perception, and haze knowledge.

## 2. Literature Review

### 2.1. Public Perception and Environmental Risk Management

As environmental issues become more complicated, new challenges appear to environmental risk management, and attention to public perception has been called upon besides traditional focus on natural science analysis. On the one hand, environmental risk management promotes institutional changes and/or policy adjustments to improve environment quality, which would change public behavior and public perception [12]. On the other hand, it is reported that public perception and value judgment among citizens are essential not only to environmental policy design and implementation, but also to social stability [13]. Thus, it is reasonable to add public perception as an indispensable factor to environmental risk management.

Numerous studies have revealed that public perception could strongly affect the level of public support for, or opposition to projects with potential environmental impacts [14,15,16]. Key influential factors are identified that may influence public perception with regarding to environmental decisions, including: the trustworthiness in the government or operating enterprises, the perceived risks, the cost perception associated with environmental risk or solutions, and the level of individual knowledge [17,18]. These factors are subjective and time varying. Besides, it is still not very clear how these factors impact public perception and facilitate the environmental risk management.

Early study of public perception can be traced back to the 1960s. Scholars define public perception as individual’s subjective cognition of various objective risks existing in the outside world, and emphasize the effects of the experience acquired by intuitive judgment and subjective understanding on their cognition [19]. Scholars find that the perception of risks is not only related to the benefit assessment of risk itself, but also to subjective factors such as voluntariness [20]. Based on the paradigm model of psychological measurement, Slovic [21] personifies the risk sources and forms a risk cognition map according to the multidimensional risk characteristics. Langford et al. [22] established a dynamic multidimensional model of risk perception based on cognitive psychology, social science, geography, and information science to capture the dynamic changes of risk perception. Nuclear energy was one of the most popular areas in risk perception study.

To clearly define and measure public perception, existing literature mainly adopts the variable of “acceptance”, which reflects the public’s subjective perception and judgment of certain activities or technologies [19,21,23]. For nuclear energy development, the level of public acceptance clearly shows the level of individual support the local construction of nuclear power plants, thus, becomes an important reference for government decision-making. Existing literature explains public acceptance of nuclear power from following factors: the risk perception of risk sources, trust in nuclear power enterprises or governments, knowledge of nuclear energy, and necessity or benefits of nuclear power development [17,18,24,25,26]. The usage of acceptance concept has been applied to other studies with regarding to public perception, such as sustainable development, climate change, and renewable energy development [23,27]. When we review existing studies on public perception of haze, we notice that these literatures mainly adopt the concept of willingness to pay to reflect perception measurement (i.e., payment for haze control, and the purchase of haze prevention products), but have seldom generated systematical discussion about the public perception from the social psychology perspective [28,29]. To fill the gap, this paper follows the research path of public acceptance.

It is worth noting, however, the concept of public acceptance may contain various acceptance levels ranging from voluntary acceptance to “reluctant acceptance” [30,31]. Based on a risk-risk trade-off scenario, the residents living next to nuclear power stations are afraid of the possible radioactive harm, but they also value the benefits of nuclear power, such as job opportunities, sufficient energy supply, mitigating climate change and others [30,31]. In contrast to “voluntary acceptance”, the public is more likely to hold an attitude of “reluctant acceptance” towards industry development with potential haze crisis. Along the same line, in this paper, people have complicated feelings about the haze pollution. They understand haze is the byproduct of the past development without considering environmental impacts. Although China started to fight against haze pollution, it takes time to fix the environmental degradation. In addition, the prosperous of the city still depends on industry development and population increase that may further worsen haze situation. The general public, in this case, probably do not really want to “accept” haze, and are likely to feel “reluctant acceptance” towards it. To clarify, this paper adopts “haze tolerance” rather than “haze acceptance” as the dependent variable. Specifically, haze tolerance refers to the public’s reluctant acceptance of the possible health risks and uncertain impacts of haze crises.

**Figure 1:**
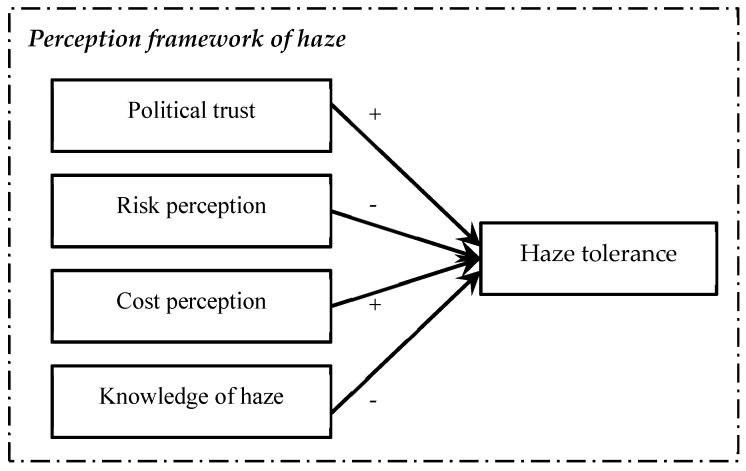
Theoretical framework of public perception towards haze.

As shown in Figure 1, haze tolerance is impacted by four factors: political trust, perceived risk, cost perception, and knowledge of haze, in which perceived risk is defined as an individual’s subjective judgment of adverse consequences of a particular hazard and threats to the environment or one’s own health, and it only describes a partial reason of haze tolerance. The following four sections will explain these factors respectively.

### 2.2. Haze Perception Model Based on Public Perception Theory

#### 2.2.1. Political Trust

The governments play an essential role in effectively managing haze risks through administrative power, policy design, risk communication, and other tools to control public emotions within tolerable limits. Therefore, the public’s trust in administrative power is an important part of haze perception. According to existing literature, political trust is the public’s basic evaluation of how well the government operates based on their expectation, specifically including the dimensions of willingness and competence, fairness, and responsiveness, outcome, and process [32,33]. Previous studies have shown that public trust in risk regulators, such as the government, can not only directly enhance public acceptance of renewable energy with uncertainty as nuclear power, but also effectively enhance public acceptance by enhancing benefit perception or reducing risk perception [34]. Especially when facing complex risks with high technical difficulty and uncertainty, the public often lacks essential knowledge and information for rational decision-making, so the risk or benefit judgment largely depends on their trust in the government [35].

In China, as the government is the most important actor of environmental governance, it has a significant impact on the public’s perceptions of environmental risks. The government has taken various policy instruments to control haze and improve air quality by eliminating highly polluting motor vehicles, adjusting and optimizing industrial structure, improving clean energy supply, and strengthening the permission of energy saving and environmental protection [36]. Existing research has shown that the public’s satisfaction with policy instruments of the government would directly influence their worries and fears about haze pollution [37]. If the public trusts the government’s willingness and capacity to control haze, they are more likely to hold the opinion that haze risks will be effectively controlled and eliminated, thus showing higher tolerance for haze. Therefore, this paper assumes that:

**Hypothesis** **1:** *People with higher political trust tend to tolerate haze pollution more.*

#### 2.2.2. Risk Perception

With the development of the public perception theory, Fischoff [38] has created the research paradigm of the psychosocial perspective, which has been widely used by scholars to explore the influencing factors of public perception towards nuclear power and in these studies risk perception only has been considered as one influencing element [17,18,24,25,26]. Risk perception is an individual’s subjective judgment of adverse consequences of a particular hazard and threats to the environment or one’s own health [19]. Previous studies have shown that risk perception has a negative relationship with the public’s support of energy technologies with uncertainty, such as the nuclear power. Specifically, if the public thinks that the development of nuclear energy would pose risks to their health, environment, and social stability, they tend to be less willing to accept the local development of nuclear power plants [17,18,39]. Also, spatial proximity and sense of place might serve as important mediating or moderating factors in risk perceptions of local nuclear power stations, thus influencing public support for new nuclear build in the local [31,40].

Regarding haze risk, scientific research shows that human exposure to PM_2.5_ can lead to chronic respiratory and cardiovascular diseases, damage in human lung tissues and defense systems, cancer and even a reduction in life expectancy [3]. Thus, if the public thinks that haze will bring health hazards to them or more haze crises in the future, they might have a higher risk perception and be less willing to tolerate haze pollution. Therefore, this paper assumes that:

**Hypothesis** **2:** *People with higher risk perception tend to tolerate haze pollution less.*

#### 2.2.3. Knowledge of Haze

Both the amount and accuracy of knowledge is crucial to people’s acceptance of risks. Because of the complexity of knowledge and technical uncertainty, it is difficult for the public to understand nuclear power scientifically and comprehensively. Many studies have found that the knowledge level of nuclear can significantly affect public acceptance [20,41]. It has been revealed that lack of knowledge or disinformation is a major factor in opposition to new technology, while substantial knowledge could help them understand the consequence of risks more objectively and hold a more rational judgment on the development of new technology [27,42]. However, some scholars found a negative relationship [26,43], and no relationship between knowledge level and nuclear power acceptance [17]. These inconsistent results might be related to the specific content of knowledge, type of risk sources, and the attributes of the sample population. Meanwhile, existing research also investigates the role of knowledge in risk acceptance within the fields of climate change, environmental protection, renewable energy, transgenic technology and so on [14,15,44].

As people usually get acquainted with negative information of haze such as the severity level of haze pollution and health hazards caused by haze, the knowledge level might have a negative relationship with people’s tolerance for haze. Therefore, this paper assumes that:

**Hypothesis** **3:** *People with more knowledge of haze tend to tolerate haze pollution less.*

#### 2.2.4. Cost Perception and Control Variables

To some extent, risk control might decrease certain benefits, which brings cost perception to the public. Huang et al. [18] revealed many benefits for local economic and social development brought by nuclear energy deployment, such as providing employment, lowering electricity prices, optimizing energy structure, and improving the environment. Local residents will think that if they oppose nuclear power construction, they will not be able to enjoy all the potential benefits from it. Considering this as high opportunity cost, they would like to be more willing to accept potential risks or nuclear technology [17,18,34,39].

In terms of haze control, the government tends to carry out specific policies to alleviate pollution by requiring the public to change their travel mode, adjusting industrial structure and eliminating high-polluted plants. For example, road transport is one of the main sources of PM_2.5_ accounting for approximately 25–30% per year in urban cities, such as Beijing [45], Shanghai [46], Guangzhou [47], Hangzhou [48], and Nanjing [49]. Thus, traffic control policies have been wildly adopted by local governments to improve air quality to the detriment of travel comfort and convenience [50]. Thus, cost perception of travelling might significantly affect the public acceptance of haze risk control [51,52,53]. Therefore, this paper assumes that:

**Hypothesis** **4:** *People with higher cost perception tend to tolerate haze pollution more.*

Additionally, existing literature states that socio-demographic features, such as gender, age, education, and others, might have effects on their acceptance levels of risk [54,55]. For instance, some scholars have found that people such as female, elder and those with long-term illnesses tend to perceive higher levels of risk and are less likely to accept nuclear power [18]. However, there is no consensus of the causal effect relationship between these social-demographic features and individual acceptance level. In this research, we control all these elements with no strong causal prediction, including gender, age, education, income, whether have smoking history or suffer from respiratory system, cardiovascular, and cerebrovascular diseases.

## 3. Research Design

### 3.1. Sampling and Data Collection

Due to fast industry development and large population density, Beijing-Tianjin-Hebei, Yangtze River Delta, and Pearl River Delta are three of the main haze pollution zones in China, with the highest PM_2.5_ concentration in Beijing, Shanghai, and Guangzhou. However, because of the difference of geographical location and energy structure, the PM_2.5_ concentration is most severe in Beijing, followed by Shanghai and Guangzhou in turn. According to the statistics of the environmental protection department in 2016, the annual average concentration of PM_2.5_ in Beijing was 73μg/m^3^, with 38 days of serious pollution [56], while the annual average concentration of PM_2.5_ in Shanghai was 45μg/ m^3^ [57], and that in Guangzhou was only 36μg/ m^3^ [58]. For better understanding of the public’s haze perception in China, we selected Beijing, Shanghai, and Guangzhou as the research sites and conducted a questionnaire survey from March to May 2016.

This study adopts the method of “stratified sampling” to collect our data. Firstly, we selected different districts in the urban and suburban areas of Beijing, Shanghai, and Guangzhou. In details, we selected Chaoyang District, Haidian District, Xicheng District, and Fengtai District in Beijing, Tianhe District, Yuexiu District, Panyu District and Nansha District in Guangzhou, and Huangpu District, Xuhui District, Jing’an District, Baoshan District, and Jiading District in Shanghai. Secondly, we randomly chose respondents in each district based on the home addresses; ten households were visited in each selected streets or communities.

As survey methods uses human data, we ensured that all subjects gave their informed consent for inclusion before they participated in the study. The study was conducted in accordance with the Declaration of Helsinki, and the protocol was approved by the Ethics Committee of the Chinese Natural Science Foundation project No. 71874098. Three questionnaire investigators were recruited to face-to-face interview respondents and help them to finish the questionnaire in each city, and finally 580 questionnaires were distributed in three cities.

After dropping the incomplete questionnaires, we got 196 valid questionnaires in Beijing, 136 in Shanghai, and 185 in Guangzhou. Among the total 517 questionnaires, 52.80 % of the respondents were male and 47.20 % were female, with an average age of 35.5 years old. 58.03% of the respondents hold a college degree. 17% of them were either smokers or suffered from respiratory, cardiovascular, and cerebrovascular diseases (see Table 1 for details). We compared our sample with the sixth national census data of three cities [59], in which male accounted for 51.79% of the total population while female accounted for 48.21%. In this sense, our research sample could well represent the population of these cities in terms of gender distribution. In addition, the average age of residents in three cities was 35.6 years old, which is quite similar with the means of 35.8 in our sample. Therefore, our sample is representative of the population in these cities as a whole.

### 3.2. Measurement Design

The existing research of risk perception mainly focuses on the field of nuclear energy, exploring the influence of trust, risk perception, benefit perception and knowledge of nuclear power on the public’s risk acceptance of nuclear power [17,18,24,25,26]. We designed our measurements according to the questionnaire of Katsuya [17] and Huang et al. [14], and modified them to conform to the specific context of haze. Our questionnaire can be divided into six parts, namely, socio-demographic characteristics, haze tolerance, political trust, risk perception, cost perception, and knowledge of haze, with a total of 31 questions (see Table 2 for details). Except for the socio-demographic characteristics, the other five parts are all in the form of Likert’s five-point scale, where “1” means strongly disagree, “3” means neutral, and “5” means strongly agree.

#### 3.2.1. Haze Tolerance

Haze tolerance refers to the public’s tolerance for haze pollution, which directly captures the public perception of haze risk. In the questionnaire, we measured people’s haze tolerance by asking about the tolerance of haze weather and their protective measures for haze days, including four questions, such as “I think haze weather is acceptable”, “I can accept going out without wearing a mask on haze days” and others.

#### 3.2.2. Political Trust

Political trust is a basic evaluative orientation towards the political system, political institutions, and their operation according to people’s normative expectations [60]. Generally speaking, the public with higher political trust tends to believe the government has the capacity to control risks and prevent harmful consequences via monitoring in real time and implementing effective policy measures. Regarding haze risk, trust in governments mainly includes two aspects: first, the open and transparent haze pollution data provided by the government; second, the governmental capacity of controlling haze. Based on the questionnaire of Katsuya [17] and Huang et al. [18], we designed four questions from these aspects to measure political trust, such as “I think the official haze pollution data are open and transparent”, “I think the government has the capacity to control haze” and others.

#### 3.2.3. Risk Perception

Human exposure to PM_2.5_ can cause chronic respiratory and cardiovascular diseases and damage human lung tissue and defense system [3]. According to Katsuya [17] and Huang et al. [18], this paper mainly measures the risk perception from the health hazards caused by haze, including six questions, such as “I feel I will have a shorter life due to haze during my life in Beijing” and others.

#### 3.2.4. Cost Perception

Usually, the government tends to control haze pollution via restricting vehicle travel or shutting down high pollution plants, which might diminish benefits such as traffic convenience and economic development. Based on the questionnaire of Katsuya [17] and Huang et al. [18], we measured cost perception of haze control from three items, that is, “haze control will bring traffic inconvenience to people’s travel”, “haze control is not conducive to local economic development” and “haze control will reduce employment opportunities”.

#### 3.2.5. Knowledge of Haze

Knowledge of haze is about how much and how accurate the individual’s understanding of haze risk is. Previous studies have shown that their level of knowledge will affect people’s attitude towards environmental risks and even facilitate the public’s pro-environmental awareness or behavior [27,61]. According to the questionnaire designed by Katsuya [17] and Huang et al. [18], we measure the public’s knowledge of haze from five questions, such as “I have substantial knowledge of haze”, “I want to get haze pollution data every day” and others. Later, the exploratory factor analysis shows that these five questions can be categorized into “knowledge level” and “knowledge acquisition intention”.

We also adopted Cronbach’s α and confirmatory factor analysis (CFA) to rest the reliability and validity of our measurements separately. According to Hinton et al. [62], “… 0.5 to 0.75 is generally accepted as indicating a moderately reliable scale, while a figure below this generally indicates a scale of low reliability”. Also, Hair et al. [63] stated that the construction of the measurement would be valid if the CFA loadings were more than 0.5. As shown in Table 2, Cronbach’s α of all measurements is above 0.67, representing high reliability. The factor loading of confirmatory factor analysis is at least 0.51, showing good validity.

## 4. Empirical Results

### 4.1. Descriptive Comparison

According to the framework of risk perception towards haze, we conducted a descriptive statistical analysis in terms of haze tolerance, political trust, risk perception, cost perception, and knowledge of haze in Beijing, Shanghai, and Guangzhou, also comparing residents’ perception of haze in these cities according to the results of ANOVA analysis.

#### 4.1.1. Haze Tolerance: Residents are Hardly Able to Tolerate Haze Risk and Have High Demand for Protective Measures, with Beijing Residents Having the Lower Level of Haze Tolerance and Protection Demand

Regarding haze tolerance, all residents have low tolerant level (shown in Table 3). As shown in Table 4, according to the ANOVA analysis of haze tolerance among three cities, compared with Guangzhou residents, residents in Beijing tolerate haze significantly less (*p* = 0.001). We attribute this difference to the different level of haze pollution in these cities. As Beijing experiences the worst haze situation, risk perception level, and haze knowledge level could be high, while makes local residents to be alert of the pollution and to have rather low acceptance rate. On the other hand, we found that the average of the variable “I can accept there is no indoor air purifier on haze days” in Beijing being the lowest as the protective measurement (compared with Shanghai: *p* = 0.007, compared with Guangzhou: *p* = 0.013), which is conflict with their low haze tolerance. This perception-behavior gap has been reported in other perception studies, though not necessary with regarding to haze pollution [64]. However, further study is needed to provide reasonable explanations.

#### 4.1.2. Political Trust: Beijing Residents have Highest Trust to the Governmental Capacity on Haze Control, while Guangzhou and Shanghai Residents have Lower Level of Trust

Table 5 describe the distribution of political trust among three cities. Shown in Table 6, Beijing residents have the highest level of trust in the official pollution data (compared with both cities: *p* = 0.000), governmental capacity on haze control (compared with both cities: *p* = 0.000), effectiveness of current control methods (compared with Shanghai: *p* = 0.000) and future control innovations (compared with both cities: *p* = 0.000). Whereas, Shanghai residents’ trust level of current haze control policies is the lowest (compared with Beijing: *p* = 0.000, compared with Guangzhou: *p* = 0.060). We attribute political trust to the government’s efforts of haze control. For example, with severe haze pollution, the Beijing government has attached great importance to air quality improvement and has contributed much personnel and financial resources to control haze in the past years. Local residents witnessed the government’s willingness and efforts in haze control, thus enhanced residents’ confidence in governmental capacity. For the other cities, compared with Guangzhou, haze pollution in Shanghai is more serious, but not serious enough for the local government to show great effort dealing with it as it has been treated in Beijing. When facing haze threats, local residents of Shanghai would be worried about the harmful consequences and dissatisfied with governmental capacity, which decrease people’s political trust.

#### 4.1.3. Risk Perception: All Residents have High Level of Risk Perception, with Guangzhou Residents Showing the Lowest

As shown in Table 7, people in these three cities are all worried about haze risk. Based on the ANOVA analysis in Table 8, Guangzhou residents have the lowest risk perception in terms of health hazards (compared with Beijing: *p* = 0.016, compared with Shanghai: *p* = 0.000), its frequent occurrence (compared with Beijing: *p* = 0.002, compared with Shanghai: *p* = 0.000), possibility to shorter life (compared with Beijing: *p* = 0.018, compared with Shanghai: *p* = 0.028), hazards to families and friends (compared with Beijing: *p* = 0.032, compared with Shanghai: *p* = 0.002) and greater harm than smoking (compared with Beijing: *p* = 0.001, compared with Shanghai: *p* = 0.003). Shanghai residents are scared about the deterioration trend of haze the most (compared with Beijing: *p* = 0.000, compared with Guangzhou: *p* = 0.007). The difference of local citizens’ risk perception towards health hazards in these three cities is closely related with the severity level of haze pollution. Also, as Beijing residents are more confident about the haze control capacity of government, they are less likely to think that the haze crisis will continue to deteriorate in the future, while Shanghai residents tend to think haze is getting worse due to their political distrust.

#### 4.1.4. Cost Perception: Residents do not Think Haze Control Would Diminish Specific Benefits, and Shanghai Residents’ Cost Perception is the Lowest while Guangzhou Residents’ is Highest

Table 9 describe the distribution of perceived cost among three cities, and all residents show less concern about the cost of haze control. According to the ANOVA analysis shown in Table 10, Guangzhou residents worried the most to possible employment opportunity lost (compared with Beijing: *p* = 0.001, compared with Shanghai: *p* = 0.000) and harm to economic development (compared with Beijing: *p* = 0.006, compared with Shanghai: *p* = 0.000). However, as shown in Table 10, the worry to potential cost is the lowest in Shanghai. The lowest cost perception of Shanghai citizens partly implies their support for the government to launch haze control measures. At the same time, Shanghai citizens have high level of risk perception, which also lead to their strong willingness to control haze at the expense of economic development or travel convenience.

#### 4.1.5. Knowledge of Haze: Residents Have Substantial Knowledge of Haze and Strong Intention to Acquire Knowledge, with Shanghai Residents Having the Strongest Acquisition Intention and Beijing Residents Having the Best Self-Evaluation of Knowledge Level

As shown in Table 11, the public has a strong will to acquire haze-related knowledge, with an overall average of 4.22. According to the ANOVA analysis in Table 12, Shanghai residents have the most positive attitude to acquiring haze pollution data (compared with Beijing: *p* = 0.046, compared with Guangzhou: *p* = 0.000), causes and health effects (compared with Beijing: *p* = 0.015, compared with Guangzhou: *p* = 0.000), and haze control strategies (compared with Guangzhou: *p* = 0.001). Regarding the self-evaluation of haze knowledge, all residents of these three cities have a good command of knowledge, with an overall average of 3.64. Based on the ANOVA analysis, compared with Guangzhou, Beijing residents have the higher level of knowledge (Learn information frequently: *p* = 0.000, Substantial knowledge: *p* = 0.008). Shanghai residents have the greatest enthusiasm for acquiring haze-related knowledge, which might be due to their risk perception. With the result that they strongly want to get more knowledge of haze to better protect themselves from the health hazards caused by haze pollution. Additionally, as the government, social media, or environmental groups often publicize and popularize haze-related knowledge, Beijing citizens have the higher self-evaluation level of haze knowledge.

### 4.2. Analysis of the Influencing Factors of Haze Tolerance

To better describe the relationship between various elements in the framework of haze risk perception, we try to further analyze the influencing factors of haze tolerance. As shown in model (1), this paper takes “tolerance of haze weather” as the dependent variable and political trust, risk perception, cost perception, and knowledge, as well as socio-economic attributes as independent variables (*X_i_*) for regression analysis. Since the dependent variable “tolerance of haze weather” is the ordinal, we adopt the ordinal logistic regression (Ologit) to analyze data, and use the ordinary least square (OLS) (In statistics, ordinary least squares (OLS) is a type of linear least squares method for estimating the unknown parameters in a linear regression model and it chooses the parameters of a linear function of a set of explanatory variables by the principle of least squares.) method as the robustness test [65].

*Risk tolerance* = α + β*X_i_* + *u* (1)

First, we do a confirmatory factor analysis for four variables of political trust, seven variables of risk perception and three variables of willingness to acquire knowledge, to reduce the dimensions and combine them into one factor separately. The coefficients of Kaiser-Meyer-Olkin test were 0.7887, 0.8855 and 0.6745 respectively, indicating that they are all very suitable for factor analysis. Additionally, we average the two variables of knowledge level to form a variable. Then, we run the model (1) and finally get the regression results as follows (shown in Table 13):

#### 4.2.1. Knowledge Level has a Negative and Significant Relationship with Haze Risk Tolerance

For the whole sample, people with more knowledge tend to be less likely to tolerate haze risks (β = −0.24, *p* < 0.1). This negative relationship is more obvious in the sample of Guangzhou, with a β coefficient of −0.57 and a p value less than 0.01. However, it does not show any significant effects for the other two cities. Existing research on risk perception stated that knowledge are the key factors to influence the public’s risk attitude and relevant behavior, but the actual relationship between knowledge level and risk acceptance is not clear: some scholars revealed the positive effects while others found negative, also some research showed no significant relationship between knowledge level and risk acceptance [17,26,43,66,67]. These inconsistent results may be related to the respondents’ knowledge content and the specific questions of the questionnaire. If the interviewees know more about the hazardous results of the risk, they are less inclined to accept such risks [26,43]. Zhang & Xue’s [37] research shows that the public who has more knowledge of haze hazards is more likely to worry and to fear about haze weather. In accordance with existing literature, our study also found that, the more the public has knowledge of haze, the more unacceptable the occurrence of haze. As most of the knowledge advertised or publicized is about the severity of current haze pollution or health hazards caused by haze, people tend to have a stronger risk perception and thus resist haze weather. Compared with residents in Beijing and Shanghai, those in Guangzhou who have a higher level of knowledge are less likely to accept haze. The current data cannot explain why knowledge levels have different impacts in each of the three cities, and we will explore it through follow-up research and relevant qualitative research.

#### 4.2.2. People with Higher Political Trust Tend to Tolerate Haze Risks More

On the whole, the more the public trusts in the government’s capacity of haze control, the higher their haze tolerance (β = 0.73, *p* < 0.01), while the positive relationship in the sample of Shanghai was slightly weakened (β = 0.38, *p* < 0.1) and more significant in the sample of Guangzhou (β = 0.73, *p* < 0.01). In previous studies, scholars found that public trust in the government, especially the capacity to control risks, can significantly improve public acceptance of risks [13,14]. Regarding haze risk, Zhang and Xue’s [37] research shows that respondents who are less satisfied with the control policies or measures are more worried and afraid of haze weather. This study also verified the positive relationship between political trust and haze risk tolerance, that is, if people trust the transparency of official pollution data and the governmental capacity to control haze, they tend to think the air quality will be improved in the future and thus could tolerate current haze weather more. The positive effect of political trust on haze risk tolerance is most significant in Guangzhou, while no significance in Beijing. Further study is needed to explore the different impacts of political trust in these three cities.

#### 4.2.3. Risk Perception Negatively Influences Haze Tolerance while Economic Cost Perception Has a Positive Effect

Risk perception and cost perception have opposite effects on haze tolerance, that is, people with stronger risk perception tend to be less likely to tolerate haze risks (β = −0.64, *p* < 0.001), while economic cost perception positively affects haze tolerance (β = 0.49, *p* < 0.001). Compared with Shanghai and Guangzhou, the influence of risk and economic cost perception is greater for Beijing residents, with the coefficient of risk perception being −1.24 (*p* < 0.001) and economic cost perception being 0.83 (*p* < 0.05). Previous studies have shown that people with higher risk perception or lower benefit perception would be less willing to accept risks [17,18,39]. Conforming to existing literature, if the respondents are more concerned about the possible health hazards of haze or less worried about the economic damage caused by haze control, they would be less inclined to accept haze weather.

In addition, the ordinal logistics model with the whole sample shows that the fact that residents in Beijing cannot tolerate haze (β = −0.74, *p* < 0.01), might be due to the severe haze pollution in Beijing. In Beijing and Guangzhou, there is a negative correlation between residents’ educational level and their tolerance. People with better education might have more knowledge of haze and understand its harmful consequences better, thus they are hardly able to tolerate haze weather. The results of the OLS model are basically consistent with those of the ordinal logistics model, which verified the robustness of our empirical results.

## 5. Conclusions

Haze pollution is the most serious environmental risk in China. As haze is harmful to people’s health, everyone has paid great attention to the severity and the control strategies of haze pollution, thus public perception is vital to managing haze risks. Based on the theory of risk perception, this paper explores the influencing factors of haze tolerance in Beijing, Shanghai, and Guangzhou from the four dimensions, namely, political trust, risk perception, cost perception, and knowledge of haze.

The results show that Beijing residents have the lowest tolerance level for haze, the highest political trust, and the best self-evaluation of knowledge. Whereas, Shanghai residents have the lowest cost perception and the strongest willingness to acquire knowledge of haze. These results are basically related to the severity level of haze pollution and the haze control efforts of the governments in three cities. At the same time, we employ the ordinal logistics model and ordinary least square method to analyze the influencing mechanism of haze tolerance:

First, political trust has positive effects on haze tolerance, and it is of great importance for the government to enhance citizens’ trust in their haze control capacity and policy transparency. As the most important role in environmental governance, the government enhances people’s pro-environmental production and lifestyle via launching environmental policies, imposing pollution taxes, popularizing environmental education and others, aiming at improving the environmental quality. Citizens’ trust in government represents their support for the various governance measures and the belief the air quality could be effectively improved within a certain period of time, so they are more tolerant of the current haze weather. As controlling haze risks to a socially acceptable level is vital to risk management, the government could enhance haze risk management via facilitating political trust. On the one hand, the practitioners are supposed to launch various effective policy instruments and promote the resources investment to control haze, showing their willingness and capacity for environmental governance. On the other hand, the government could try to put the public back into the policy-making process, enhancing the transparency and their understanding of haze control policies, thus facilitating local residents’ trust in the government.

Second, risk perception is a key factor to affect public tolerance of haze and the effectiveness of risk management. The results show that people with stronger risk perception tend to be more worried about the health hazards caused by haze or future deterioration of haze, thus less likely to tolerate haze weather. Additionally, if the public has more knowledge of the sources, formation mechanism and impacts of haze pollution, they might know more about the harmful consequences of haze and be less tolerant. Therefore, it is necessary for the government to publicize the knowledge of haze pollution, such as the potential health hazards of haze, to help the public to acquire objective and scientific knowledge and diminish unnecessary worries. Also, the practitioners could popularize the protective measures to prevent the public from haze pollution, to avoid exaggerating risk perception of haze.

However, this study also has some limitations. First, our sample only comes from Beijing, Shanghai, and Guangzhou, all of which are economic developed cities. The lack of economic developing cities and large sample size pose a challenge to the external validity of our results to some extent. Second, the questionnaire survey is a subjective “self-report” by the interviewees, which might inevitably deviate from their real behavior. Also, as people are highly likely to overestimate their capacity in self-reported assessment, our self-rated measurement of knowledge might generate threats to validity and reliability. Third, the study found that the influencing factors of public haze tolerance in different cities are slightly different, but the existing data cannot explain these differences in detail. Usually, the results obtained through statistical regression need to be further supplemented and explained by qualitative research. In the future, we will enlarge our sample size with more cities of different economic development and natural environment, introduce methods of measuring actual behavior, and adopt a qualitative study, to further explore the influencing mechanism of haze tolerance.

## Figures and Tables

**Table 1:** Distribution of sample socio-demographics.

Characteristics		Frequency	Percentage (%)
Gender	Male	274	53.00
	Female	243	47.00
Age	14–19	25	4.84
	20–29	246	47.58
	30–39	102	19.73
	40–49	36	6.96
	50–59	34	6.58
	60–69	51	9.86
	70–87	23	4.45
Monthly income	<2000	52	10.06
	2000–5000	129	24.95
	5001–10,000	179	34.62
	10,001–20,000	106	20.50
	>20,000	51	9.86
Education	Middle school or below	34	6.58
	High school	69	13.35
	College	300	58.03
	Master’s or above	114	22.05
Diseases	Yes	431	83.37
	No	86	16.63
Smoker	Yes	428	82.79
	No	89	17.21

Source: analysis of the questionnaires.

**Table 2:** Questionnaire of risk perception towards haze and reliability and validity tests.

Variables		Measurements	Cronbach’s α	λ (Factor Loading)
Haze tolerance	Haze weather	I think haze weather is acceptable	0.6786	0.5123
	Protective measures	I can accept going out without a mask on haze days		0.6100
		I can accept outdoor exercise on haze days		0.5915
		I can accept there is no indoor air purifier on haze days		0.5520
Political trust	Control capacity	I think the government has the capacity to control haze	0.8559	0.7204
	Pollution data	I think the official haze pollution data are open and transparent		0.7989
	Control measures	I think the current haze control policies and measures are appropriate and trustworthy		0.8247
		I think the government will facilitate the innovation of haze control policies and measures in the future		0.6956
Risk perception	Health hazards	I’m worried about the health hazards caused by haze	0.8744	0.6329
		I think haze will frequently appear in Beijing/Shanghai/Guangzhou and endanger people’s health		0.6189
		I will have a shorter life due to haze during my stay in Beijing/Shanghai/Guangzhou		0.8651
		I’m very scared of the haze in Beijing/Shanghai/Guangzhou		0.8248
		My families and friends will suffer from respiratory diseases, asthma, cardiovascular and cerebrovascular diseases, and cancer due to haze		0.7473
		I think haze is more harmful to human health than smoking		0.6356
	Deterioration trend	I think the haze in Beijing/Shanghai/Guangzhou will become increasingly serious in the future		0.6353
Cost perception	Economic cost	Haze control will reduce employment opportunities	0.8371	0.7246
		Haze control is not conducive to local economic development		0.8332
	Traffic cost	Haze control will bring traffic inconvenience to people’s travel		0.7624
Knowledge of haze	Acquisition intention	I want to get haze pollution data every day	0.8425	0.6419
		I want to know the causes of haze and its impact on human health		0.8914
		I want to know the current control strategy of haze pollution		0.8771
	Knowledge level	I often learn about haze information by surfing the Internet, watching TV, or reading newspapers	0.6863	0.6334
		I have substantial knowledge of haze (source, formation mechanism, and impacts)		0.6334

**Table 3:** Distribution of tolerance degree in Beijing, Shanghai, and Guangzhou.

		Beijing			Shanghai			Guangzhou			Total
		Distribution	Average	S.D.	Distribution	Average	S.D.	Distribution	Average	S.D.	Total Average
Haze tolerance	Disagree	90.16%	1.48	0.71	80.88%	1.61	0.95	88.65%	1.78	0.74	1.62
	Neutral	8.2%			15.44%			8.65%			
	Agree	1.64%			3.68%			2.7%			
No mask	Disagree	56.53%	2.39	1.05	43.39%	2.46	1.15	61.95%	2.26	1.00	2.37
	Neutral	28.26%			44.12%			25%			
	Agree	15.22%			12.5%			13.04%			
Outdoor exercise	Disagree	78.26%	1.88	0.89	78.67%	1.66	0.94	71.89%	2.04	0.95	1.86
	Neutral	15.76%			17.65%			19.46%			
	Agree	5.98%			3.68%			8.65%			
No indoor air purifier	Disagree	26.78%	3.05	1.05	40.44%	2.68	1.21	36.21%	2.73	0.98	2.82
	Neutral	34.43%			36.76%			43.24%			
	Agree	38.8%			22.8%			20.54%			

**Table 4:** ANOVA analysis of tolerance degree among Beijing, Shanghai, and Guangzhou.

	Beijing vs. Shanghai	Beijing vs. Guangzhou	Shanghai vs. Guangzhou
Haze tolerance	0.1487	0.3030 ****	0.1543
No mask	0.0803	−0.125	−0.2053
Outdoor exercise	−0.2188	0.1628	0.3816 ****
No indoor air purifier	−0.3725 ***	−0.3195 **	0.0530

Notes: * *p* < 0.1, ** *p* < 0.05, *** *p* < 0.01, **** *p* < 0.001.

**Table 5:** Distribution of political trust in Beijing, Shanghai, and Guangzhou.

		Beijing			Shanghai			Guangzhou			Total
		Distribution	Average	SD	Distribution	Average	SD	Distribution	Average	SD	Total Average
Open and transparent pollution data	Disagree	4.4%	3.16	1.11	21.32%	2.65	1.24	14.05%	2.58	0.98	2.80
	Neutral	19.23%			32.35%			30.81%			
	Agree	76.37%			46.32%			55.13%			
Strong control capacity	Disagree	28.42%	3.64	0.98	44.85%	3.10	1.37	31.35%	3.03	1.01	3.26
	Neutral	33.88%			32.35%			42.7%			
	Agree	37.7%			22.79%			25.94%			
Appropriate control measures	Disagree	11.41%	3.12	1.09	34.56%	2.65	1.22	27.57%	2.90	0.99	2.89
	Neutral	26.63%			28.68%			40%			
	Agree	61.96%			36.76%			32.44%			
Future control innovation	Disagree	27.03%	3.87	0.84	44.12%	3.32	1.22	45.4%	3.48	0.94	3.56
	Neutral	32.43%			33.09%			38.38%			
	Agree	40.54%			22.8%			16.21%			

**Table 6:** ANOVA analysis of political trust among Beijing, Shanghai, and Guangzhou.

	Beijing vs. Shanghai	Beijing vs. Guangzhou	Shanghai vs. Guangzhou
Open and transparent pollution data	−0.5456 ****	−0.5784 ****	−0.0328
Strong control capacity	−0.5456 ****	−0.6088 ****	−0.0632
Appropriate control measures	−0.5112 ****	−0.2229	0.2883 *
Future control innovation	−0.5804 ***	−0.3980 ****	0.1824

Notes: * *p* < 0.1, ** *p* < 0.05, *** *p* < 0.01, **** *p* < 0.001.

**Table 7:** Distribution of perceived risk in Beijing, Shanghai, and Guangzhou.

		Beijing			Shanghai			Guangzhou			Total
		Distribution	Average	SD	Distribution	Average	SD	Distribution	Average	SD	Total Average
Health hazards	Disagree	3.78%	4.27	0.89	2.21%	4.49	0.79	5.4%	4.02	0.85	4.26
	Neutral	8.65%			11.76%			15.68%			
	Agree	87.57%			86.03%			78.92%			
Frequent occurrence	Disagree	9.14%	4.10	1.04	5.89%	4.36	0.94	17.84%	3.72	1.21	4.06
	Neutral	8.6%			26.47%			12.97%			
	Agree	82.26%			67.65%			69.19%			
Shorter life	Disagree	8.06%	4.12	0.96	6.62%	4.14	1.06	14.75%	3.83	1.04	4.03
	Neutral	10.22%			21.32%			14.75%			
	Agree	81.72%			72.06%			70.5%			
Scaring haze	Disagree	4.86%			5.89%			9.73%			
	Neutral	18.92%	4.03	0.90	26.47%	3.99	1.01	22.16%	3.84	0.97	3.95
	Agree	76.21%			67.65%			68.11%			
Hazards to families and friends	Disagree	8.7%			2.21%			13.51%			
	Neutral	14.13%	4.03	1.05	27.21%	4.16	0.93	15.14%	3.76	0.98	3.98
	Agree	77.18%			70.59%			71.35%			
More severe haze	Disagree	20.22%			5.15%			14.59%			
	Neutral	26.78%	3.55	1.16	22.79%	4.06	0.96	24.86%	3.68	1.02	3.76
	Agree	53.01%			72.06%			60.54%			
Greater harm than smoking	Disagree	8.11%	4.10	0.95	8.82%	4.09	1.10	9.73%	3.71	0.95	3.97
	Neutral	10.81%			20.59%			30.81%			
	Agree	81.08%			70.59%			59.54%			

**Table 8:** ANOVA analysis of perceived risk among Beijing, Shanghai, and Guangzhou.

	Beijing vs. Shanghai	Beijing vs. Guangzhou	Shanghai vs. Guangzhou
Health hazards	0.2185 *	−0.2485 **	−0.4671 ****
Frequent occurrence	0.2512	−0.3832 ***	−0.6344 ****
Shorter life	0.0095	−0. 2931 **	−0.3027 **
Scaring haze	−0.0550	−0.1946	−0.1396
Hazards to families and friends	0.1232	−0.2650 **	−0.3882 ***
More severe haze	0.4932 ****	0.1238	−0.3694 ***
Greater harm than smoking	−0.0125	−0.3892 ****	−0.3767 ***

Notes: * *p* < 0.1, ** *p* < 0.05, *** *p* < 0.01, **** *p* < 0.001.

**Table 9:** Distribution of perceived cost in Beijing, Shanghai, and Guangzhou.

		Beijing			Shanghai			Guangzhou			Total
		Distribution	Average	SD	Distribution	Average	SD	Distribution	Average	SD	Total Average
Employment opportunity	Disagree	76.54%	1.98	1.05	75.73%	1.74	1.10	59.46%	2.39	1.12	2.04
	Neutral	12.29%			16.91%			23.24%			
	Agree	11.17%			7.36%			17.29%			
Economic development	Disagree	84.24%	1.79	0.95	81.62%	1.57	0.99	70.27%	2.11	1.05	1.82
	Neutral	8.7%			13.97%			17.3%			
	Agree	7.06%			4.41%			12.43%			
Traffic convenience	Disagree	87.91%	1.71	0.96	88.23%	1.38	0.78	81.09%	1.87	1.00	1.65
	Neutral	6.04%			9.56%			9.73%			
	Agree	6.05%			2.21%			9.19%			

**Table 10:** ANOVA analysis of perceived cost among Beijing, Shanghai, and Guangzhou.

	Beijing vs. Shanghai	Beijing vs. Guangzhou	Shanghai vs. Guangzhou
Employment opportunity	−0.2690 *	0.4059 ****	0.6749 ****
Economic development	−0.2521 *	0.3200 ***	0.5722 ****
Traffic convenience	−0.3253 ***	0. 1615	0.4868 ****

Notes: * *p* < 0.1, ** *p* < 0.05, *** *p* < 0.01, **** *p* < 0.001.

**Table 11:** Distribution of knowledge in Beijing, Shanghai, and Guangzhou.

		Beijing			Shanghai			Guangzhou			Total
		Distribution	Average	SD	Distribution	Average	SD	Distribution	Average	SD	Total Average
Pollution data	Disagree	7.7%	4.02	0.98	7.35%	4.24	1.10	12.97%	3.65	0.99	3.97
	Neutral	13.74%			17.65%			23.24%			
	Agree	78.58%			75%			63.79%			
Causes and healthy impacts	Disagree	2.17%	4.33	0.74	2.21%	4.59	0.78	3.24%	4.19	0.83	4.37
	Neutral	8.15%			11.76%			10.27%			
	Agree	89.67%			86.03%			86.49%			
Haze control strategy	Disagree	2.19%	4.32	0.76	2.21%	4.51	0.82	3.78%	4.16	0.87	4.33
	Neutral	9.29%			13.97%			13.51%			
	Agree	88.53%			83.83%			82.7%			
Learn information frequently	Disagree	5.5%			13.23%			16.21%			
	Neutral	21.98%	3.96	0.93	24.26%	3.82	1.20	32.97%	3.49	1.00	3.76
	Agree	72.53%			62.5%			50.81%			
Substantial knowledge	Disagree	14.21%			13.23%			17.39%			
	Neutral	25.14%	3.64	1.01	24.26%	3.57	1.11	38.04%	3.34	0.86	3.52
	Agree	60.66%			62.5%			44.57%			

**Table 12:** ANOVA analysis of knowledge among Beijing, Shanghai, and Guangzhou.

	Beijing vs. Shanghai	Beijing vs. Guangzhou	Shanghai vs. Guangzhou
Pollution data	0.2768 **	−0.3624 ***	−0.6392 ****
Causes and healthy impacts	0.2529 **	−0.1369	−0.3898 ****
Haze control strategy	0.1814	−0. 1656	−0.3470 ****
Learn information frequently	−0.1495	−0.4751 ****	−0.3255 **
Substantial knowledge	−0.0884	−0.3079 ***	−0.2194

Notes: * *p* < 0.1, ** *p* < 0.05, *** *p* < 0.01, **** *p* < 0.001.

**Table 13:** Influencing mechanism of haze tolerance.

		Risk Tolerance _Whole Sample		Risk Tolerance _Beijing		Risk Tolerance _Shanghai		Risk Tolerance _Guangzhou	
		Ologit	OLS	Ologit	OLS	Ologit	OLS	Ologit	OLS
Knowledge acquisition intention		−0.17 (−1.34)	−0.05 (−1.05)	−0.22 (−0.68)	−0.05 (−0.67)	−0.41 (−1.64)	−0.16 (−1.61)	0.09 (0.49)	0.02 (0.41)
Knowledge level		−0.24 * (−1.88)	−0.06 (−1.34)	0.15 (0.50)	0.05 (0.64)	0.05 (0.20)	0.05 (0.54)	−0.57 *** (−2.65)	−0.15 ** (−2.24)
Political trust		0.41 *** (3.14)	0.11 *** (2.60)	0.41 (1.32)	0.07 (1.03)	0.38 * (1.61)	0.05 (0.64)	0.73 *** (3.02)	0.22 *** (3.32)
Risk perception		−0.64 **** (−4.98)	−0.22 **** (−5.09)	−1.24 **** (−4.09)	−0.34 **** (−4.82)	−0.77 *** (−3.20)	−0.26 *** (−2.89)	−0.31 (−1.41)	−0.09 (−1.32)
Economic cost perception		0.49 **** (3.51)	0.16 *** (3.20)	0.83 ** (2.22)	0.17 * (1.74)	0.66 ** (2.32)	0.19 (1.57)	0.34 (1.52)	0.11 (1.57)
Traffic cost perception		−0.23 (−1.58)	−0.06 (−1.11)	−0.41 (−1.20)	−0.09 (−1.00)	−0.10 (−0.31)	0.11 (0.83)	−0.07 (−0.31)	−0.04 (−0.54)
Gender		−0.18 (−0.82)	−0.06 (−0.83)	−0.43 (−0.97)	−0.10 (−0.84)	0.01 (0.01)	−0.03 (−0.15)	−0.25 (0.70)	−0.08 (−0.74)
Age	21–40	0.52 (1.30)	0.15 (1.05)	−0.48 (−0.27)	−0.07 (−0.15)	−	−	0.95 ** (2.01)	0.27 * (1.88)
	41–60	0.42 (0.79)	0.17 (0.91)	−2.03 (−1.09)	−0.45 (−0.92)	0.76 (0.97)	0.48 * (1.68)	2.02 ** (2.19)	0.57 * (1.89)
	>60	0.65 (1.08)	0.17 (0.82)	−0.63 (−0.34)	−0.15 ** (−2.12)	−0.74 (−0.53)	−0.08 (−0.15)	−	−
Education	High school or below	−0.66 (−0.97)	−0.28 (−1.19)	−0.69 (−0.79)	−0.21 (−0.90)	−	−	−3.21 * (−1.89)	−1.12 ** (−2.09)
	College	−0.54 (−0.83)	−0.25 (−1.14)	−1.38 (−1.58)	−0.42 * (−1.77)	0.65 (0.75)	0.20 (0.63)	−2.50 * (1.91)	−0.90 ** (−2.23)
	Master’s or above	−0.50 (−0.74)	−0.20 (−0.88)	−2.07 ** (−2.01)	−0.58 ** (−2.12)	0.49 (0.51)	0.25 (0.71)	−2.50 * (1.89)	−0.89 ** (−2.21)
Income		−0.13 (−1.56)	−0.05 (−1.64)	0.61 *** (2.63)	0.15 ** (2.42)	−0.43 ** (−2.54)	−2.49	−0.09 (−0.73)	−0.03 (−0.67)
Smoking		−0.44 (−1.54)	−0.11 (−1.16)	−0.46 (−0.70)	−0.09 (−0.60)	0.23 (0.43)	0.40	−0.98 * (−1.94)	−0.30 ** (−1.99)
Diseases		−0.19 (−0.60)	−0.06 (−0.59)	−0.17 (−0.28)	−0.01 (−0.09)	−0.06 (−0.10)	−0.18	0.06 (0.08)	0.01 (−0.02)
Beijing		−0.74 *** (−2.63)	−0.20 ** (−2.10)	−	−	−	−	−	−
Shanghai		−0.07 (−0.27)	0.09 (0.98)	−	−	−	−	−	−
Cons_		−	2.03 **** (6.31)	−	0.97 (1.40)	−	1.53 *** (2.74)	−	2.96 **** (6.06)
$R^{2}$		0.1373	0.2184	0.1962	0.2934	0.1962	0.3170	0.1366	0.2424
N		517		196		136		185	

Notes: * *p* < 0.1, ** *p* < 0.05, *** *p* < 0.01, **** *p* < 0.001.
